# The Usefulness of Antigen Testing in Predicting Contagiousness in COVID-19

**DOI:** 10.1128/spectrum.01962-21

**Published:** 2022-03-29

**Authors:** Tulio J. Lopera, Juan Carlos Alzate-Ángel, Francisco J. Díaz, María T. Rugeles, Wbeimar Aguilar-Jiménez

**Affiliations:** a Grupo Inmunovirología, Universidad de Antioquia, Medellín, Antioquia, Colombia; University of Cincinnati

**Keywords:** SARS-CoV-2, COVID-19, antigen detection test, viral isolation, RT-PCR

## Abstract

Increasing the diagnostic capacity for COVID-19 (SARS-CoV-2 infection) is required to improve case detection, reduce COVID-19 expansion, and boost the world economy. Rapid antigen detection tests are less expensive and easier to implement, but their diagnostic performance has been questioned compared to reverse transcription-PCR (RT-PCR). Here, we evaluate the performance of the Standard Q COVID-19 antigen test for diagnosing SARS-CoV-2 infection and predicting contagiousness compared to RT-PCR and viral culture, respectively. The antigen test was 100.0% specific but only 40.9% sensitive for diagnosing infection compared to RT-PCR. Interestingly, SARS-CoV-2 contagiousness is highly unlikely with a negative antigen test since it exhibited a negative predictive value of 99.9% compared to viral culture. Furthermore, a cycle threshold (*C_T_*) value of 18.1 in RT-PCR was shown to be the one that best predicts contagiousness (area under the curve [AUC], 97.6%). Thus, screening people with antigen testing is a good approach to prevent SARS-CoV-2 contagion and allow returning to daily activities.

**IMPORTANCE** The importance of our results is the excellent agreement between the Standard Q COVID-19 antigen test and the viral culture, indicating that it is important as a marker of contagiousness. Due to its high positive predictive value in situations of a high prevalence of infection, positive results do not require confirmation with another test. Likewise, its high negative predictive value for contagiousness makes possible to use this test as a criterion to discharge patients in isolation and screen people moving into environments that could facilitate the transmission of the virus. Screening people with antigen testing is a good approach to prevent SARS-CoV-2 contagion and allow returning to daily activities.

## INTRODUCTION

Coronavirus disease 2019 (COVID-19) caused by SARS-CoV-2 has resulted in a global health crisis that requires substantial efforts worldwide to increase the diagnosis capacity and improve detection of cases by using inexpensive, easy, and rapid testing (1). On the other hand, the economic reopening requires a diagnostic test before returning to daily activities. Therefore, a negative result in a diagnostic test has become the entrance door to many countries or to other activities that involve some risk of transmission (2).

Reverse transcription-PCR (RT-PCR) has become the gold standard test for SARS-CoV-2 detection due to its high sensitivity and specificity (3). However, it has been observed that RT-PCR remains positive long beyond the period of contagiousness. Therefore, the return to daily activities is delayed, generating unnecessary restrictions for patients in the convalescent period. In these patients, viral RNA can be detected in low loads (approximate cycle threshold [*C_T_*] value of >24 in RT-PCR), and contagiousness is unlikely. In addition, the contagiousness depends on high viral load, not days since symptom onset or severity (4, 5). For this reason, classical techniques such as viral isolation are used as a better predictor of viral infectivity. However, these methodologies are expensive, risky, and time-consuming.

In 2020, antigen detection (point-of-care [POC]) tests for SARS-CoV-2 were developed (6). The first antigen test approved for diagnostic use in Colombia was the Standard Q COVID-19 Ag test (SD Biosensor, Republic of Korea), which is based on immunochromatography and detects the nucleocapsid (N) antigen of SARS-CoV-2 using monoclonal antibodies (7). Antigen detection tests have shown desirable diagnostic characteristics such as reasonable specificity, fast execution, and easy processing (7, 8).

The sensitivity and specificity of diagnostic tests are essential parameters used to guide decision making regarding diagnostic tests. However, antigen tests are considered inferior to RT-PCR because their sensitivity for detecting SARS-CoV-2 is lower (9, 10), likely since the performance of antigen tests can be affected by the days since symptom onset (DSO) and viral load during infection (8, 11, 12). Likewise, the usefulness of the antigen test for predicting contagiousness remains to be determined (13). Thus, we aimed to evaluate the performance of a rapid antigen detection test approved for use in Colombia using RT-PCR and viral culture as the gold standard tests for diagnosing infection and contagiousness, respectively.

## RESULTS

### Demographic and clinical characteristics of the population.

**FIG 1:**
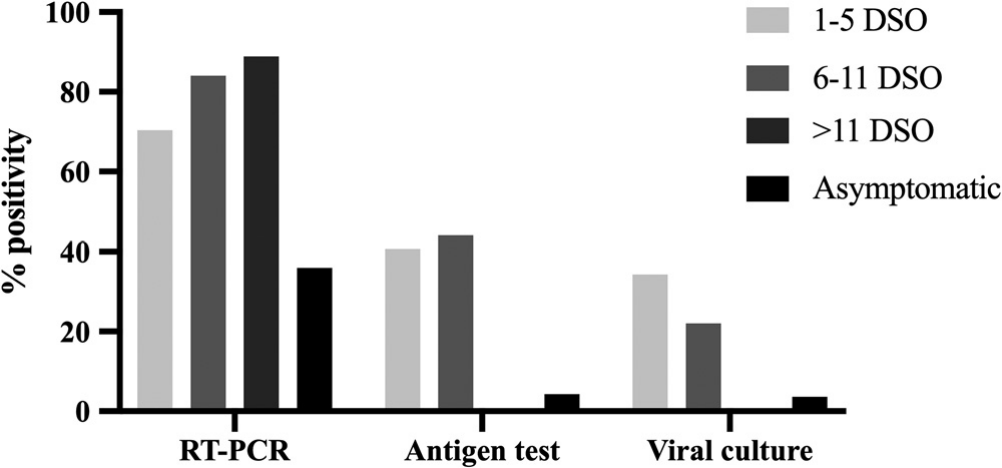
Positivity (%) of each test according to DSO or asymptomatic condition. The figure shows the percentage of positive results obtained by RT-PCR, antigen test, and viral culture in patients who were on 1 to 5 days since symptom onset (DSO), 6 to 11 DSO, or >11 DSO and people who were asymptomatic individuals.

In this study, 306 nasopharyngeal samples were analyzed. All samples were made blind and coded before the analyses. The median age of subjects was 38 years (range 18 to 96 years), and 58.9% were female. Considering that the performance of diagnostic tests may fluctuate depending on DSO, we allocated the samples among three diagnostic scenarios: (i) 108 samples came from subjects with 1 to 5 DSO, (ii) 50 came from individuals with 6 to 11 DSO, and (iii) 139 samples were from SARS-CoV-2-exposed subjects without symptoms (some symptomatic subjects also disclosed previous close contact with a person diagnosed with COVID-19). In addition, 9 samples were included from individuals with a previous diagnosis and more than 11 DSO in order to find the time frame in which isolation and antigen detection were possible (Fig. 1).

The percentage of positivity in RT-PCR was higher than 70% in individuals with symptoms but below 40% in asymptomatic ones. In contrast, less than 40% positivity by antigen test and viral culture was seen in subjects with either 1 to 5 or 6 to 11 DSO, and no positivity was observed in subjects with more than 11 DSO. As expected, the positivity in viral culture decreased as days with symptoms increased (Fig. 1).

The definitive diagnosis of infection was made by RT-PCR. In total, 176 samples were SARS-CoV-2 RT-PCR positive (57.5% positivity), most of them from outpatients who resolved COVID-19 at home, and seven were from hospitalized patients. The clinical and demographic characteristics of subjects in the study were recorded in a search for associations with the result of the antigen test (see Table S2 in the supplemental material) and viral culture (Table S3).

### Performance of antigen test in the diagnosis of infection.

People who tested positive by antigen test were older (age median [range] = 45.5 [20.0 to 96.0] years) than people who tested negative (age median [range] = 37 [18.0 to 84.0] years), and a higher proportion of them exhibited cough, fever, odynophagia, dyspnea, fatigue, conjunctivitis, headache, and anosmia or ageusia, compared to those with a negative antigen test. No differences in gender between subjects with positive and those with negative antigen tests were observed (Table S2).

The performance of the SARS-CoV-2 antigen test for infection diagnosis was evaluated with RT-PCR as the reference standard. From 176 RT-PCR-positive specimens, 72 were positive by antigen test, revealing a low sensitivity (40.9%, 95% confidence interval [CI] = 33.6 to 48.6%). No RT-PCR-negative sample was positive in the antigen test, demonstrating high specificity for SARS-CoV-2 infection (100%; 95% CI = 96.4% to 100%). Indeed, the probability of COVID-19 was high in the participants with a positive result in the antigen test (positive predictive value [PPV] 100%, 95% CI = 93.7 to 100%). Nonetheless, the concordance between the antigen test and RT-PCR was generally low, with a kappa index of 0.37 (95% CI = 0.29 to 0.44).

We observed the best performance of antigen test in samples from patients with 1 to 5 DSO, in which the diagnostic sensitivity was 57.9% (95% CI = 46.0% to 68.9%), the specificity was 100% (95% CI = 86.7% to 100%), and concordance with the RT-PCR using the kappa index was 0.44 (95% CI = 0.32 to 0.58). In patients between 6 and 11 DSO, the sensitivity and specificity of the antigen test were 52.4% (95% CI = 36.6% to 67.7%) and 100% (95% CI = 59.8% to 100%), respectively. In asymptomatic subjects, the performance of the antigen test decreased, showing a poor sensitivity (12.0%; 95% CI = 5.0% to 25.0%), although the specificity was similar to the other categories (100%; 95% CI = 94.8 to 100%) (Table 1).

**TABLE 1:** Performance of antigen test, viral culture, and RT-PCR in diagnosis of infection and prediction of contagiousness of SARS-CoV-2^*a*^

Scenario	Sensitivity, % (95% CI)	Specificity, % (95% CI)	PPV (95% CI)	NPV (95% CI)	LR^+^ (95% CI)	LR^−^ (95% CI)
Comparison, antigen test vs RT-PCR (reference)						
All subjects	40.9 (33.6, 48.6)	100 (96.4, 100)	100 (93.68, 100)	55.0 (48.0, 61.0)	107.31 (6.7, 1,716.2)	0.59 (0.52, 0.7)
1–5 DSO	57.9 (46.0, 68.9)	100 (86.7, 100)	1.00 (0.89, 1.00)	50.0 (38.0, 61.0)	38.14 (2.4, 601.05)	0.42 (0.32, 0.5)
6–11 DSO	52.4 (36.6, 67.7)	100 (59.7, 100)	100 (81.0, 100)	28.0 (13.0, 48.0)	9.41 (0.6, 141.4)	0.47 (0.3, 0.6)
Asymptomatic	12.00 (5.0, 25.00)	100 (94.8, 100)	100 (51.0, 100)	66.0 (58.0, 74.0)	22.94 (1.3, 398.9)	0.88 (0.8, 0.9)
Comparison, antigen test vs viral culture (reference)						
All subjects	96.2 (85.9, 99.3)	91.0 (87.0, 94.0)	70.0 (58.0, 80.0)	99.14 (96.6, 99.9)	11.6 (7.7, 17.5)	0.0 (0.0, 0.2)
1–5 DSO	97.0 (84.0, 99.0)	88.0 (78.0, 94.0)	81.0 (66.0, 91.0)	98.0 (90.0, 99.0)	8.6 (4.5, 16.6)	0.0 (0.0, 0.2)
6–11 DSO	90.0 (57.0, 99.0)	69.0 (52.0, 82.0)	45.0 (25.0, 67.0)	96.0 (79.0, 99.0)	3.0 (1.8, 4.9)	0.1 (0.0, 0.9)
Asymptomatic	100 (46.3, 100)	99.0 (95.0, 99.0)	83.0 (36.0, 99.0)	100 (96.0, 100)	134.0 (19.0, 944.3)	0.1 (0.0, 1.2)
Comparison, RT-PCR vs viral culture (reference)						
All subjects	100 (91.0, 100)	51.0 (45.0, 57.0)	30.0 (23.0, 37.0)	100 (96.0, 100)	2.1 (1.8, 2.3)	0.0 (0.0, 0.3)
1–5 DSO	100 (88.0, 100)	45.0 (33.0, 57.0)	48.0 (37.0, 60.0)	100 (86.0, 100)	1.8 (1.5, 2.2)	0.0 (0.0, 0.5)
6–11 DSO	100 (67.0, 100)	20.0 (9.0, 36.0)	26.0 (14.0, 0.42)	100 (59.0, 100)	1.25 (1.1, 1.5)	0.2 (0.0, 3.2)
Asymptomatic	100 (46.0, 100)	66.0 (57.0, 74.0)	10.0 (3.0, 22.0)	100 (94.0, 100)	3.0 (2.3, 3.8)	0.1 (0.0, 1.8)

a PPV, positive predictive value; NPV, negative predictive value; LR+, positive Likelihood-Ratio; LR−, negative Likelihood-Ratio.

### Positivity of antigen test and viral culture is linked to *C_T_* value in the RT-PCR.

**FIG 2:**
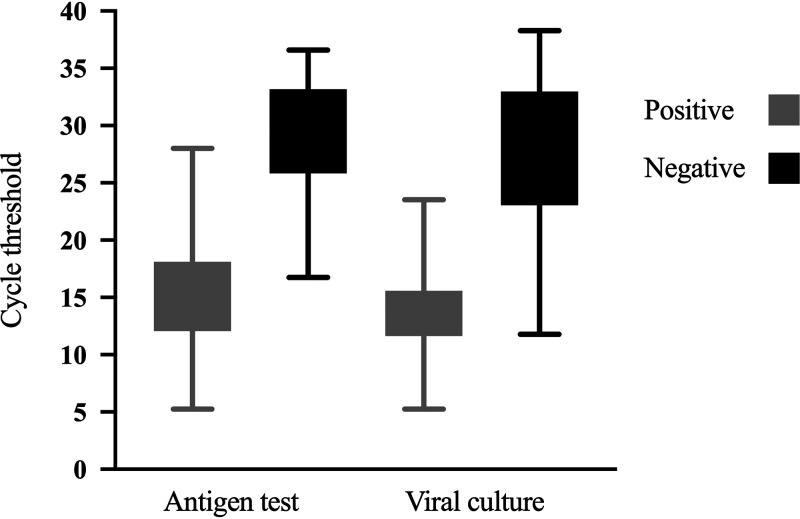
*C_T_* values according to results in the antigen test and viral culture. The figure displays the distribution according to cycle threshold (*C_T_*) values obtained in RT-PCR of positive and negative results of antigen test and viral culture in patients with SARS-CoV-2 infection.

Considering that the *C_T_* value in the RT-PCR is an indicator of viral load, we explored the distribution of *C_T_* values between positive and negative samples by antigen test and viral culture (Fig. 2). Interestingly, the *C_T_* values among both antigen test and viral culture had a similar distribution. The positive antigen test had a median *C_T_* value (interquartile range) of 15.2 (12.1 to 18.1), and positive viral culture had a median *C_T_* value (interquartile range) of 13.7 (11.6 to 15.6) while the negative antigen test had a median *C_T_* value (interquartile range) of 28.93 (16.7 to 36.6), and the negative viral culture had a median *C_T_* value (interquartile range) of 27.47 (11.8 to 36.6) (Fig. 2). Indeed, out of 65 samples with a *C_T_* of <20 by RT-PCR, 62 (95.4%) were also positive by antigen test, and 51 (78.5%) were also positive by viral culture.

### Prediction of contagiousness.

All samples were assessed by viral culture and antigen test, even when the RT-PCR was negative. SARS-CoV-2 was isolated in 53 (17.3%) of 306 samples (30% when considering only the 176 samples positive by RT-PCR). The clinical and demographic characteristics of the population were recorded in search of associations with the result of viral culture and thus its usefulness to predict contagiousness. People with a positive culture test had a similar age (median age [range] = 44.0 [20.0 to 96.0] years) as people without SARS-CoV-2 isolation (median age [range] = 38 [18.0 to 86.0] years). In addition, people with a positive culture had a significantly higher frequency of fever, cough, odynophagia, fatigue, dyspnea, headache, and anosmia or ageusia, compared to those with a negative culture (Table S3).

We subsequently explored the usefulness of antigen tests in predicting contagiousness, as measured by viral isolation *in vitro* (Table 1). The concordance between viral culture and antigen test was acceptable, with a kappa index of 0.77 (95% CI = 0.68 to 0.85). Of 53 viral culture-positive samples, 51 were also positive in the antigen test, showing a high sensitivity (96.2%; 95% CI = 85.9% to 99.3%). In addition, we observed a high negative predictive value of 99.1% (95% CI = 96.6% to 99.9%) in predicting contagiousness (Table 1). Likewise, we found that the antigen test predicts contagiousness more accurately 1 to 5 DSO (kappa index of 0.82, 95% CI = 0.71 to 0.93) than 6 to 11 DSO (kappa index of 0.44, 95% CI = 0.21 to 0.67).

Asymptomatic subjects who were RT-PCR positive usually had a low viral load (median *C_T_* [interquartile range] = 29.6 [26.79 to 33.44]); therefore, most samples were negative by antigen test (95.6%) and viral culture (96.4%). Nevertheless, in this group, the sensitivity of the antigen test for contagiousness was high (100% [95% CI = 46.29% to 100%]) (Table 1).

We carried out a multiple logistic regression model (Table S3) with variables such as the *C_T_* value in RT-PCR and those showing a crude association with antigen test or viral culture results (Tables S1 and S2). Although positivity in antigen test was associated with positivity in viral culture (crude odds ratio [OR] = 134.79 [30.45, 596.64]), the association was not statistically significant after covariate adjustment (adjusted OR = 5.79 [0.5, 66.85]) (Table S3). In contrast, the categorized *C_T_* value was associated with the positivity in viral culture after adjustment. Indeed, the samples with a *C_T_* value of <20 and <15 had adjusted ORs of 25.07 (2.27, 277.23) and 290.45 (17.19, 4,907.16), respectively (Table S3).

### Predicting contagiousness by RT-PCR.

**FIG 3:**
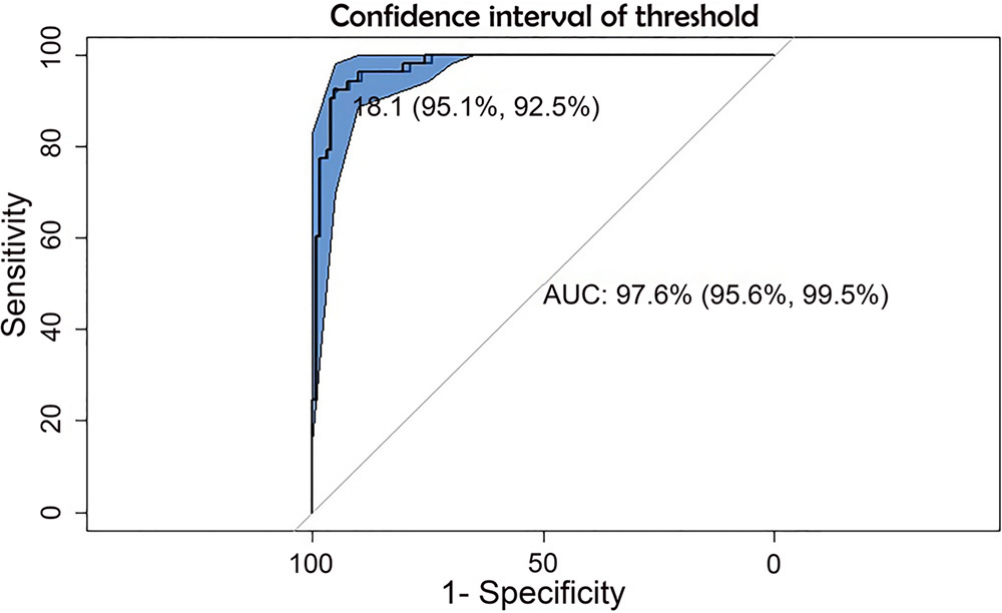
ROC curve and AUC to calculate the discriminatory *C_T_* value in contagiousness. The receiver operating characteristic (ROC) curve shows the *C_T_* value where sensitivity and specificity are higher than 95% to predict the contagiousness using viral culture as reference. Area under the curve (AUC), 97.6% (95.6% to 99.5%).

According to previous findings, the RT-PCR *C_T_* value, as an indicator of viral load, could help in predicting infection. Therefore, we analyzed the *C_T_* value to find the value that best predicts a positive result in viral culture. An analysis of a receiver operating characteristic (ROC) curve was performed, obtaining a *C_T_* value of 18.1 as the best predictor of a positive result in viral culture, with an area under the curve (AUC) of 97.6% (95% CI = 95.6% to 99.5%) (Fig. 3). Our results also showed that the highest *C_T_* value where a viral isolate was obtained was 23.5 (Fig. 2).

## DISCUSSION

We evaluated the Standard Q antigen test performance in diagnosing SARS-CoV-2 infection and predicting contagiousness. This rapid and simple antigen test is widely used in Colombia and other countries, especially in high-prevalence populations; its sensitivity has varied widely across different evaluations (14–19). In this study, the sensitivity for infection diagnosis in all samples was low, 40.9% (95% CI = 33.6 to 48.6%), much lower than the 84% sensitivity reported by the manufacturer (8). Nevertheless, other authors have reported significant variation in sensitivity ranging between 42.9% (20), 70.7% (21), and 98.3% (15).

This variability seems to be only partially explained by the DSO at sampling because the sensitivity of the antigen test in subjects with 1 to 5 DSO increased up to 57%, whereas in subjects with 6 to 11 DSO the sensitivity was 52%. Indeed, other studies have reported similar sensitivity values in subjects with ≤14 DSO (55.4%) (19). On the other hand, our sampling population consists mostly of outpatients, who usually have mild disease and consequently lower viral load. Actually, we observed similar sensitivity as the 63% reported in patients with the same disease spectrum (22). Indeed, when we evaluated the performance of the antigen test compared to the *C_T_* in RT-PCR, the proportion of positive antigen tests was 62/65 (95.4%) in samples with a *C_T_* of <20, in concordance with other reports indicating around 95% sensitivity of the antigen test in samples with high viral load (*C_T_* less than 22.5) (19, 23).

Although the sensitivity of the antigen test was low, its high specificity and high positive predictive value indicate that a positive result in this test provides reliable evidence of SARS-CoV-2 infection; conversely, a negative result does not rule out infection. However, additional testing in subjects with a negative antigen test could be unnecessary if we are interested in detecting patients who are shedding virus and therefore are a threat to those close to them.

Remarkably, the antigen test and a positive viral culture had similar performances; in both cases, the distribution of *C_T_* values had a median of <20. Indeed, the detection limit for the Standard Q antigen test was 3.12 × 10^2.2^ 50% tissue culture infective doses (TCID_50_)/mL or ∼6.92 log RNA copies (data not shown), which is in agreement with the minimum viral loads (5.4 to 7.0 log RNA copies) found in samples with viral isolation (24, 25). Whereas there were no specific symptoms typical of COVID-19 or DSO related to the probability of viral isolation, the antigen test showed a specificity of 91% to predict viral infectivity; from the 306 samples, only 2 were discordant between antigen test and viral culture results. In addition, our findings are consistent with the study by Yamayoshi et al. (23), who found, in samples with a *C_T_* value of <22.5, similar results in the antigen test and viral isolation (11/11 were positive in antigen test and 8/11 in viral culture). Also, the study by Pekosz et al. (26) reported that the antigen test correlates with viral culture in VeroE6/TMPRSS2 cells of SARS-CoV-2 in 27/28 samples assessed. Although our cell model was unmodified VeroE6 cells, the concordance between antigen test and viral culture was acceptable. On the other hand, Huang et al. (27), using the same cell model, found a notable concordance with high viral load, which is in agreement with our results. Therefore, these results suggest that a negative antigen test is a promising tool to discharge patients from quarantine because of the low probability of transmitting the virus.

Despite some authors having reported viral isolation in culture up to a *C_T_* value close to 32 (22, 28, 29), most studies have associated viral isolation with a *C_T_* of 18 to 24 (5.4 to 7.0 log RNA copies) (24, 25). Although it is well known that the *C_T_* value is intrinsically variable (22, 30, 31), due to the conditions of the RT-PCR, the fluorescence threshold, the fluorochrome used, the gene target, and the virus lineage (12), RT-PCR is useful in the prediction of contagiousness at least in samples with high viral load (4, 5, 22). Our study indicates that the *C_T_* value that could predict more confidently the result in viral culture is 18.1, with a progressive decrease in the possibility of contagiousness above this *C_T_* value. Hence, we propose that a *C_T_* value higher than 23.5 could be a safe threshold of contagiousness since it was the highest *C_T_* in which we could isolate the virus in culture. On the other hand, our main highlight result is the antigen test performance, which targets the conserved nucleocapsid protein, as a more consistent and cost-beneficial method of assessing contagiousness than any other diagnostic tool.

In this study, the time of exposure was not controlled since it was reported by each individual, considered from the last contact with someone with a confirmed COVID-19 diagnosis. Due to this, many individuals did not know precisely the time of contact with the virus. Additionally, the data correspond to a study of diagnostic tests in which the prevalence of infection is given by the proportion of infected and uninfected subjects who participated in the study, which could not be an exact reflection of the actual prevalence in the population. Strikingly, a recent study by Currie et al. (32) analyzing only RT-PCR-positive individuals found that the antigen test had a similar positive predictive value (71%) in the prediction of contagiousness assessed by viral culture. Further studies should include a serial sampling of the participants in other prevalence scenarios to improve the scope of the results.

### Conclusion.

This study demonstrated that the Standard Q COVID-19 antigen test has excellent concordance with viral culture, indicating that it can be used as a marker of contagiousness. Due to its high positive predictive value in situations of a high prevalence of infection, positive results do not require confirmation with another test. Likewise, its high negative predictive value for contagiousness underlines its use as a criterion to discharge patients in isolation and for screening people in environments that could facilitate viral transmission.

## MATERIALS AND METHODS

### Population.

A sample size of at least 250 specimens with a ratio of positive to negative of 1:1 was calculated, anticipating a 97% specificity according to previous reports (14, 33), a probability of type I error of 5%, and a precision of 3%. The inclusion criteria to participate in the study were adults (≥18 years) with suspicion of SARS-CoV-2 infection by either clinical or epidemiological criteria (clinical symptoms or recent exposure to a confirmed case, respectively). The SARS-CoV-2 diagnosis is indicated in patients with recent (before 11 days) symptom onset compatible with acute respiratory infection and in individuals with an epidemiological nexus/contact confirmed with SARS-CoV-2-positive patients before 11 days after the contact (34). We analyzed 306 nasopharyngeal samples from 282 ambulatory subjects (some were sampled more than once as part of their follow-up) recruited at the Grupo Inmunovirología, University of Antioquia, Medellín, Colombia, between September 2020 and January 2021. We excluded pediatric patients and individuals who were unwilling to provide their written consent. The study was designed and conducted following the Declaration of Helsinki and Colombia legislation (Ministry of Health resolution 008430 of 1993). It was approved by the Ethics Committee of the Universidad de Antioquia (Act. 004, 02-04-2020). After explanation of the project and clarification of doubts about the research, all included subjects signed the informed consent. The collected biological material was encoded to ensure privacy.

### Sample collection.

Nasopharyngeal swabs collected on viral transport medium were obtained following the Centers for Disease Control and Prevention (CDC) recommendations (35). The nasopharyngeal samples were maintained at 4°C for 4 to 84 h before processing. The epidemiological and demographic data were collected from each subject filling out the official form for reporting acute respiratory infection by SARS-CoV-2 (National Institute of Health, Colombia) (36).

### Antigen test.

We used the Standard Q COVID-19 Ag test (SD Biosensor, Republic of Korea), following the manufacturer’s recommendations (8). Briefly, 350 μL of the viral transport medium was mixed for 1 to 2 min with the antigen test lysing reagent. Then, 4 drops were added to the cassette, and after 30 min, the test was interpreted as positive, negative, or invalid. The antigen tests were evaluated independently by two laboratory technicians blind to the RT-PCR and viral culture results. In cases of discrepancy, the antigen test was repeated with the same sample.

### Real-time RT-PCR.

Viral RNA extraction was performed from 300 μL viral transport medium using the column-based Quick-RNA viral kit (Zymo Research, Orange, CA) following the manufacturer’s instructions. SARS-CoV-2 viral RNA was detected using the Luna Universal Probe one-step reverse transcription-quantitative PCR (RT-qPCR) kit (New England Biolabs, MA, USA). The reaction mixture included 7 μL of viral RNA, the oligonucleotides and probe for the E gene, and the conditions reported in the Berlin real-time RT-PCR protocol v2 (15) with a thermal modification in reverse transcription (55°C for 18 min) and in the alignment/extension step (60°C for 30 s), according to the one-step RT-qPCR kit manufacturer’s recommendations.

In addition, human RNase P gene transcripts were detected as an internal control and for evaluation of the quality of the sample, as previously recommended (37). The RT-PCRs were carried out in a CFX-96 Bio-Rad thermal cycler (Bio-Rad, CA, USA). Tests were performed in parallel with a negative control (sample replaced by water) and a positive control (RNA from virus isolated at the University of Antioquia) (38).

### Viral culture.

Viral cultures were carried out in a biosafety level 3 (BSL-3) laboratory. Approximately 100 μL of the viral transport medium was dissolved in 250 μL of Dulbecco modified Eagle medium (DMEM) culture medium supplemented with 2% fetal bovine serum and 1% penicillin-streptomycin. This solution was inoculated into monolayers of 1 × 10^5^ VeroE6 cells in 12-well plates and incubated for 90 min at 37°C with 5% CO_2_. Subsequently, the inoculum was removed, and 1.5 mL of DMEM culture medium supplemented with 2% fetal bovine serum and 1% penicillin-streptomycin was added. The culture was kept at 37°C with 5% CO_2_ for 5 days. The monolayer was observed daily for a cytopathic effect (CPE; observed as rounding and detachment of infected cells) indicative of SARS-CoV-2 infection. After CPE observation, the supernatants were harvested and stored at −80°C. For confirmation purposes, some samples with or without CPE were evaluated for the presence of SARS-CoV-2 by indirect immunofluorescence (38) or by qRT-PCR on cell supernatants; all of these samples showed a perfect correlation between the presence of CPE and detection of the virus in the cell culture (see Table S1 in the supplemental material).

### Statistical analysis.

The Standard Q COVID-19 Ag test was evaluated using the sensitivity, specificity, predictive value, and likelihood ratio calculated using GraphPad Prism (version 9; CA, USA), R v4.1.0, and the Integrated Development Environment RStudio. To explore variables associated with the result of each test, bivariate analyses were carried out, and according to statistical (*P* value < 0.05) and plausibility criteria, they were included in a binary logistic regression model. Receiver operating characteristic (ROC) curves were constructed to discriminate the best cycle threshold (*C_T_*) value predicting contagiousness.
