# Supplementary material for: The Usefulness of Antigen Testing in Predicting Contagiousness in COVID-19
Source: Microbiol Spectr. 2022 Mar 29;10(2):e01962-21. doi: 10.1128/spectrum.01962-21 (PMC9045251; doi:10.1128/spectrum.01962-21)
Supplement: SUPPLEMENTAL FILE 1 — Supplemental material. Download SPECTRUM01962-21_Supp_1_seq13.pdf, PDF file, 0.1 MB [file spectrum01962-21_supp_1_seq13.pdf]

Manuscript**: The usefulness of antigen testing in predicting contagiousness in COVID-19**

Appendix Table (Supplements).

Table S1. Demographic characteristics of the population according to antigen tests result.

		**Antigen test (absolute frequency (%))**		**p-value**
		**Negative (n = 234)**	**Positive (n = 72)**	
**Variable**				
**Sex**	**Female**	136 (58.1%)	44 (61.1%)	**0.652**
	**Male**	98 (41.9%)	28 (38.9%)	
**Age (years)***		37.0 [18.0, 84.0]	45.5 [20.0, 96.0]	**0.001684**
**Cough**	**No**	177 (75.6%)	37 (51.4%)	**8.691e-05**
	**Yes**	57 (24.4%)	35 (48.6%)	
**Fever**	**No**	207 (88.5%)	45 (62.5%)	**4.344e-07**
	**Yes**	27 (11.5%)	27 (37.5%)	
**Odynophagia**	**No**	194 (82.9%)	36 (50.0%)	**1.594e-08**
	**Yes**	40 (17.1%)	36 (50.0%)	
**Dyspnea**	**No**	211 (90.2%)	50 (69.4%)	**1.409e-05**
	**Yes**	23 (9.8%)	22 (30.6%)	
**Fatigue**	**No**	179 (76.5%)	29 (40.3%)	**8.417e-09**
	**Yes**	55 (23.5%)	43 (59.7%)	
**Rhinorrhea**	**No**	209 (89.3%)	58 (80.6%)	**0.05126**
	**Yes**	25 (10.7%)	14 (19.4%)	
**Conjunctivitis****	**No**	232 (99.1%)	67 (93.1%)	**0.009017**
	**Yes**	2 (0.9%)	5 (6.9%)	
**Headache**	**No**	178 (76.1%)	33 (45.8%)	**1.242e-06**
	**Yes**	56 (23.9%)	39 (54.2%)	
**Diarrhea**	**No**	216 (92.3%)	63 (87.5%)	**0.2085**
	**Yes**	18 (7.7%)	9 (12.5%)	
**Anosmia and/or ageusia**	**No**	212 (90.6%)	46 (63.9%)	**5.047e-08**
	Yes	22 (9.4%)	26 (36.1%)	

* Median (range) ** Fisher test

Table S2. Demographic characteristics of the population according to antigen tests result.

		Viral culture (absolute frequency (%))		p value
		**Negative (n = 253)**	**Positive (n = 53)**	
**Variable**				
**Sex**	**Female**	149 (58.9%)	31.0 (58.5%)	**0.9568**
	**Male**	104 (41.1%)	22.0 (41.5%)	
**Age (years)***		38.0 [18.0, 86.0]	44.0 [20.0, 96.0]	**0.06841**
**Cough**	**No**	187 (73.9%)	27 (50.9%)	**0.0009**
	**Si**	66 (26.1%)	26 (49.1%)	
**Fever**	**No**	221 (87.4%)	31 (58.5%)	**5.398e-07**
	**Si**	32 (12.6%)	22 (41.5%)	
**Odynophagia**	**No**	205 (81.0%)	25 (47.2%)	**2.133e-07**
	**Si**	48 (19.0%)	28 (52.8%)	
**Dyspnea**	**No**	227 (89.7%)	34 (64.2%)	**1.755e-06**
	**Si**	26 (10.3%)	19 (35.8%)	
**Fatigue**	**No**	188 (74.3%)	20 (37.7%)	**2.116e-07**
	**Si**	65.0 (25.7%)	33 (62.3%)	
**Rhinorrhea**	**No**	224 (88.5%)	43 (81.1%)	**0.1416**
	**Si**	29 (11.5%)	10 (18.9%)	
**Conjunctivitis****	**No**	249 (98.4%)	50 (94.3%)	**0.1028**
	**Si**	4 (1.6%)	3 (5.7%)	
**Headache**	**No**	188 (74.3%)	23 (43.4%)	**9.749e-06**
	**Si**	65 (25.7%)	30 (56.6%)	
**Diarrhea**	**No**	233 (92.1%)	46 (86.8%)	**0.2827**
	**Si**	20 (7.9%)	7 (13.2%)	
**Anosmia and/or ageusia**	**No**	221 (87.4%)	37 (69.8%)	**0.0014**
	Yes	32.0 (12.6%)	16.0 (30.2%)	

* Median (range) ** Fisher test

Table S3. Logistic regression for the prediction of contagiousness during COVID-19

		Coefficient	Crude OR (CI95%)	Adjusted OR (CI95%)	p-value
	**(Intercept)**	-18.56			
**Ct value**	**≥ 20**	REF			
	**≥ 15 and < 20**	3.22	68.83 (13.95,339.55)	25.07 (2.27,277.23)	0.009
	**< 15**	5.67	1091.5 (148.57,8018.91)	290.45 (17.19,4907.16)	< 0.001
**Antigen test**	**Negative**	REF			
	**Positive**	1.75	134.79 (30.45,596.64)	5.79 (0.5,66.85)	0.16
**RT-PCR**	**Negative**	REF			
	**Positive**	14.34	6744082.23 (0,Inf)	1700015.17 (0,Inf)	0.995
**Days from onset of symptoms**	**Asymptomatic**	REF			
	**1 – 5**	-0.38	8.6 (3.11,23.84)	0.68 (0.06,7.39)	0.75
	**6 – 11**	-1.55	3.55 (1.13,11.19)	0.21 (0.02,2.74)	0.234
	**> 11**	-16.65	0 (0,Inf)	0 (0,Inf)	0.993
**Cough**	**No**	REF			
	**Yes**	0.12	1.74 (0.91,3.32)	1.14 (0.22,5.93)	0.88
**Fever**	**No**	REF			
	**Yes**	0.81	3.19 (1.58,6.45)	2.26 (0.39,13.26)	0.365
**Odynophagia**	**No**	REF			
	**Yes**	-0.05	3.65 (1.86,7.15)	0.94 (0.18,5)	0.946
**Dyspnea**	**No**	REF			
	**Yes**	1.10	3.13 (1.5,6.53)	3.02 (0.55,16.46)	0.202
**Fatigue**	**No**	REF			
	**Yes**	-0.09	3.08 (1.59,5.97)	0.91 (0.13,6.21)	0.924
**Headache**	**No**	REF			
	**Yes**	0.10	2.61 (1.36,5.01)	1.11 (0.21,5.74)	0.904
**Anosmia and/or ageusia**	**No**	REF			
	**Yes**	-0.47	1.47 (0.72,3)	0.62 (0.12,3.09)	0.56
